# Classic Case Report of Donohue Syndrome (Leprechaunism; OMIM ∗246200): The Impact of Consanguineous Mating

**DOI:** 10.1097/MD.0000000000002710

**Published:** 2016-02-12

**Authors:** Yousif Nijim, Youssef Awni, Amin Adawi, Abdalla Bowirrat

**Affiliations:** From the Pediatric and Neonatal Department, EMMS Nazareth—The Nazareth Hospital, Galilee Medical School—Bar-Ilan University (YN); Orthopedic Medicine, Medical Consulting Center, Nazareth Towers, General Medical Services “Sheruti Briut Clalit,” Galilee Medical School—Bar-Ilan University (YA); Pediatric and Neonatal Department, EMMS Nazareth—The Nazareth Hospital, Galilee Medical School, Galilee Medical School—Bar-Ilan University (AA); and Clinical Neuroscience, Neuropsychopharmacology & Population Genetics, Research Center, EMMS Hospital, Nazareth—The Nazareth Hospital (AB), Israel.

## Abstract

Donohue syndrome ([DS]; leprechaunism) describes a genetic autosomal recessive disorder that results from the presence of homozygous or compound heterozygous mutations in the insulin receptor gene (*INSR*; 19p13.3–p13.2).

Donohue syndrome is associated with a fatal congenital form of dwarfism with features of intrauterine and postnatal growth retardation, exaggerated hyperglycemia with hyperinsulinism and dysmorphic abnormalities.

We present a case of DS owing to the rarity of this syndrome (1 case in every million births). We discuss how the disease presents, its genetic underpinning, and its prevention.

The case was encountered in an Arab male born on 1 September, 2014, for consanguineous parents. The delivery was via cesarean section at 37 weeks gestation due to severe intrauterine growth restriction and nonprogress labor term. The patient was admitted to the Neonatal Intensive Care Unit due to infection, and jaundice. Dysmorphic features, abnormalities of the craniofacial region, low birth weight, skin abnormalities, abdominal distension and hypertrichosis were observed. Laboratory examinations showed, hyperinsulinism, increased C-peptide, thrombocytopenia, leucopenia, and anemia.

The diagnosis of DS was done based on the combinations of typical dysmorphic characteristics, clinical evaluation, supported by genetic analysis and exaggerated biochemical results. Genetic diagnosis of DS was performed through analysis of DNA via polymerase chain reaction (PCR). A qualitative real-time PCR was used, to monitor the amplification of a targeted DNA molecule during the PCR. Other technique using sequencing of the *INSR* gene, which permits genetic diagnosis, counseling, and antenatal diagnoses in subsequent pregnancies, were also performed.

Treatment of DS is supportive and requires the combined efforts of a multidisciplinary team, which include pediatricians, endocrinologists, dermatologists, and other health care professionals. Currently, treatment with recombinant insulin-like growth factor 1 demonstrates effectiveness, and a combination treatment with insulin-like growth factor binding protein 3 resulted in an increased lifespan.

There is a scarcity of genetic information on DS among the Arab population. Consanguinity is one of underlying reasons for the appearance of rare genetic disorders. Inbreeding has long been considered a controversial phenomenon. Genetic counseling and overwhelming the alertness of the negative consequences of consanguinity on public health are warranted.

## INTRODUCTION

The most common forms of diabetes mellitus are due to insulin resistance, for example, a reduced or weak response to the effects of insulin beyond its receptor. Cases of diabetes due to an impaired insulin receptor or an absent insulin receptor are rare. Here we describe a case of leprechunism, a genetic disorder secondary to congenital lack of the insulin receptor. Owing to the rarity of this condition, its unique genetic underpinnings, and the role of consanguinity and cultural norms that lead to the risk for such a disorder to occur, we present a case and discuss its implications.

## CASE REPORT

We report a case study of a 4-month-old male child of healthy, consanguineous parents living in urban environment after receiving full informed consent of the parents. The child was born on September 1, 2014 to a 26-year-old healthy mother at EMMS Nazareth Hospital. The delivery was via cesarean section at 37 weeks gestation due to severe intrauterine growth restriction and nonprogress labor term.

Apgar score at 1 minute after birth was 9, and at 5 minutes after birth was 10. Birth weight was 1290gr, head circumference was 31.3 cm, length = 36.3 cm, pulse = 138, breathing rate = 67/minute, blood pressure: systolic = 64, diastolic = 21, and blood saturation = 96.

The newborn was admitted to the Neonatal Intensive Care Unit (NICU) at the age of 1 day due to severe asymmetric IUGR, suspected infection with fever, and observed jaundice. He received immediately oxygen enrichment and antibiotic injection. During his admission at the NICU, various laboratory examinations were conducted including CBC, electrolytes, biochemistry, and several blood cultures. The laboratory findings revealed the following results: Hyperinsulinism (5560.4 pmol/L), neonatal hypoglycemia (24 mg/dL) and hyperglycemia (170 mg/dL), hypocholesterolemia (50 mg/dL), hypotriglyceridemia (36 mg/dL), cholestatic jaundice (direct bilirubin = 4.6 mg/dL; indirect bilirubin = 6.5 mg/dL), level of C-Peptide (2747 pmol/L), hypokalemia (2.8 mEq/L), neonatal thrombocytopenia (as low as 38,000/microL), leucopenia (as low as 2990/microL) and anemia of prematurity. Further investigations showed Urosepsis due to Enterobacter, right sided hydronephrosis and Hydrocele.

Clinical examinations for whole body systems were performed: chest X-Rays, cranial ultrasound, and heart echocardiogram which were all intact. Moreover, neurological examination was normal: the plantar grasp reflex, Moro reflex, and sucking reflex.

Dysmorphic features of the child were extremely abnormal, showing the following characteristics: Emaciation, lipoatrophy, genitomegaly and other distinctive characteristics including abnormalities of the craniofacial region with elfin facies, low birth weight and skin abnormalities, abdominal distension, hypertrichosis, and large low-set ears were observed clearly.

On the light of these findings, treatment was given which included: packed cells transfusion, platelet transfusion, antibiotic injections, and recombinant human insulin-like growth factor 1 (rh-IGF1) treatment was started because of poor weight gain. Several days after his admission the child's condition stabilized and he was discharged from the hospital with the recommendation of follow up in outpatient clinic.

**FIGURE 1:**
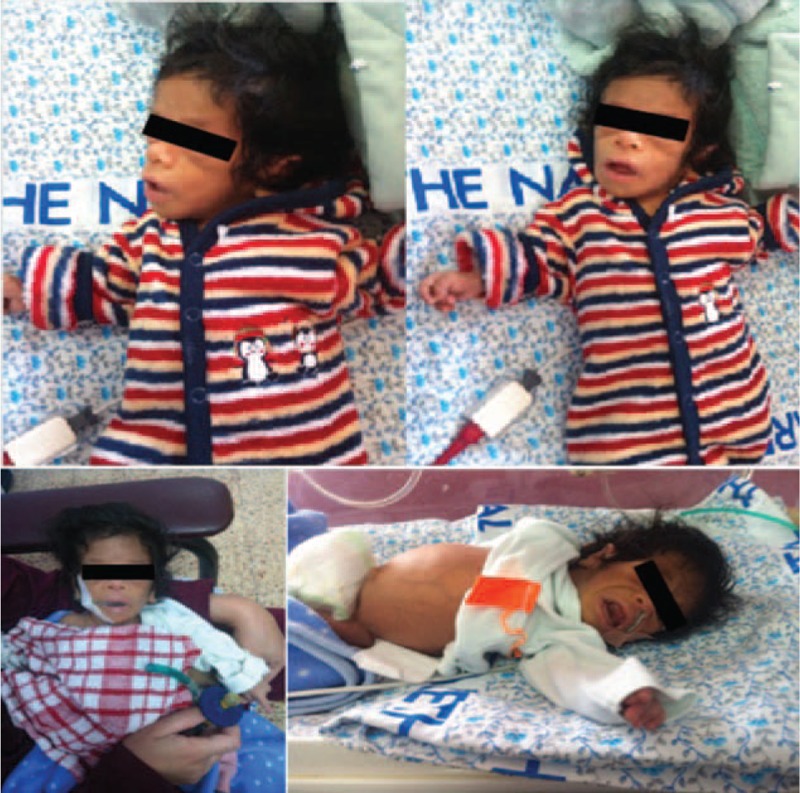
Dysmorphic features of the child were extremely abnormal, showing the following characteristics: Emaciation, lipoatrophy, genitomegaly other distinctive characteristics, including abnormalities of the craniofacial region with elfin facies, low birth weight and skin abnormalities, abdominal distension, hypertrichosis, and large low-set ears were observed clearly.

Meanwhile, on the basis of typical dysmorphic characteristics and laboratory findings especially the exaggerated hyperinsulinaemia, we suspected Donohue syndrome ([DS]; leprechaunism; OMIM 246200) (Figure 1), which is the most severe form of inherited insulin-resistance disorders. In our case, it is worth to mention that the patient was a result of consanguineous mating and the fact that in the majority of previous cases of DS are results of consanguineous mating, strengthen our suspicion of DS.^1^

**FIGURE 2:**
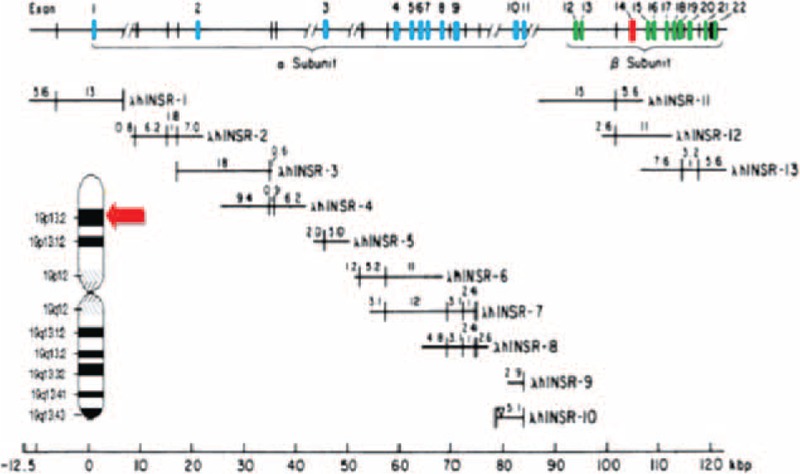
Map of the human *INSR*. Insulin receptor gene is composed of 2 extracellular alpha subunits that bind insulin and 2 beta subunits that span the plasma membrane and have an intracellular tyrosine kinase domain. The alpha and beta subunits are encoded by a single *INSR* gene found on the short arm of chromosome 19, locus p13.3 to p13.2. The gene spans >120 kilobase pairs (kbp) and has 22 exons. All introns interrupt protein coding regions of the gene. The 11 exons (exons 1–11) encoding the α subunit of the receptor are dispersed over >90 kbp, whereas the 11 exons (exons 12–22) encoding the β subunits are located together in a region of approximately 30 kbp. *INSR* = insulin receptor gene

Therefore, DNA analysis of the child and his parents was examined. After a couple of weeks, we received the following genetic findings: both parents were carriers of a heterozygous deletion encompassing exon 14 of the insulin receptor gene (*INSR*) gene (Figure 2). Homozygous mutations in the *INSR* affect the region encoding the tyrosine kinase domain or affect the insulin-binding domain of the receptor. A qualitative real-time PCR was used, which is basically real-time polymerase chain reaction that is a laboratory technique of molecular biology based on the polymerase chain reaction (PCR). This technique monitors the amplification of a targeted DNA molecule during the PCR, that is, in real-time, and not at its end, as in conventional PCR. Real-time PCR can be used quantitatively—quantitative real-time PCR (as in our case) or semiquantitatively. Furthermore, the result of the child's genetic analysis was a homozygous deletion of exon 14 on the *INSR* gene after conducting sequencing analysis. The genetic results beside the dysmorphic characteristics and laboratory findings confirm the diagnosis of DS in this child.

After 4 months of continuous follow-up in outpatient clinic and hospitalizations the patient arrived to our NICU in a critical condition presenting the following symptoms: paleness, tachycardia, high fever (39.6°C), and distended abdomen. Sepsis was suspected and immediate antibiotic treatment was administered. Day later, the patient's condition deteriorated notably and signs of bradicardia and eventually cardiac arrest appeared due to septicaemia. All efforts to resuscitate the patient were in van and death was confirmed at 30/12/2014.

## DISCUSSION

Donohue syndrome (OMIM 246200) is a genetic autosomal recessive disorder which results from the presence of homozygous or compound heterozygous mutations in the *INSR* (19p13.3–p13.2), that produce a total or near-total absence of functional insulin receptors.^2–4^

In 1948, Donohue^5^ first identified the physical and clinical findings of unusual craniofacial features, hirsutism, and various endocrine disturbances in a newborn female who was born to consanguineous parents. Six years later, a second sibling was born to the same consanguineous couple who had the same dysmorphic phenotype. Interest in this syndrome was aroused after the descriptive term “Leprechaunism” was suggested by the author.^5^

The characteristics of DS encompass a variety of distinguishing physical features and metabolic symptoms, such as severe intrauterine and postnatal growth retardation, multiple endocrine dysfunctions, hypertrichosis, virilization, emaciation, acanthosis nigricans, lipoatrophy, genitomegaly, postprandial hyperglycemia, fasting hypoglycemia, insulin resistance, hyperinsulinemia, and eventual development of ketoacidosis. Affected infants may also have distinctive characteristics, including abnormalities of the craniofacial region with elfin facies, low birth weight and skin abnormalities, and large low-set ears. It is worth to mention that many of the symptoms associated with DS differ in their severity and variation in expression from patient-to-patient might occur.^6,7^

Donohue syndrome is an extremely rare and fatal congenital form of dwarfism with less than 1 case in every million births. In reported cases, DS has occurred twice as often in females as in males (F2:M1).^8^

Donohue syndrome caused by mutations in the *INSR* resulting in either defects in insulin binding domain or receptor autophosphorylation and tyrosine kinase activity.^9–11^ Mutations in the *INSR* gene cause several inherited insulin-resistance disorders with paradoxical hypoglycaemia, which range from mild to severe. The syndromes of inherited insulin resistance actually form a wide clinical variety and many of these disorders are associated with diverse endocrine, metabolic, and genetic conditions and may reveal distinct phenotypic features.^12^

The human insulin receptor (*INSR*) (also known as CD220) is a transmembrane heterotetramer membrane glycoprotein receptor composed of 2 extracellular alpha subunits that bind insulin and 2 beta subunits that span the plasma membrane and have an intracellular tyrosine kinase domain, that is activated by insulin, thus a cascade of events is initiated, encompassing autophosphorylation, phosphorylation of cellular protein substrates, glucose transport, and glycogen synthesis. The alpha and beta subunits are encoded by a single *INSR* gene found on the short arm of chromosome 19, locus p13.3 to p13.2. The gene spans >120 kilobase pairs (kbp) and has 22 exons. All introns break off protein-coding segment of the gene. The 11 exons (exons 1–11) encoding the α subunits of the receptor are dispersed over >90 kbp, whereas the 11 exons (exons 12–22) encoding the β subunits are located together in a region of approximately 30 kbp.^13,14^

CD220 plays a crucial role in the regulation of glucose homeostasis, cell growth, differentiation, and apoptosis- functional processes that under degenerate environment may cause a plethora of clinical manifestations, such as diabetes mellitus and cancer.^13,14^

The isolation and characterization of cDNA clones encoding the human insulin receptor indicate that the α and β subunits of the insulin receptor are derived by proteolytic processing of a common 1382 amino acid preproreceptor.^13,14^

The diagnosis of DS depends on the combinations of typical dysmorphic characteristics, clinical evaluation, supported by biochemical results of exaggerated hyperinsulinaemia (usually >1000 pmol/l and sometimes in excess of 50,000 pmol/l) and genetic analysis. Prenatal diagnosis can be done through analysis of DNA obtain via amniocentesis. DNA collected from amniotic cells is analyzed via polymerase chain reaction (PCR). In DS, PCR is used to identify mutations to the insulin receptor gene. A qualitative real-time PCR is usually used, which is basically real-time polymerase chain reaction that is a laboratory technique of molecular biology based on the PCR. This technique monitors the amplification of a targeted DNA molecule during the PCR, that is, in real-time, and not at its end, as in conventional PCR. Real-time PCR can be used quantitatively—Quantitative real-time PCR (as in our case) or semiquantitatively. Sequencing of the *INSR* gene, now available on either a research or diagnostic basis in the United Kingdom,^5,6,15^ permits genetic diagnosis, counseling, and antenatal diagnosis in subsequent pregnancies.

Differential diagnosis of these disorders that share mutations in the *INSR* gene, include type-A insulin resistance syndrome, Rabson Mendenhall syndrome and DS. These disorders represent a continuum or spectrum of diseases. Rabson Mendenhall syndrome is considered a moderate form in severity between DS (which is fatal before age 2) and type-A insulin resistance syndrome (which is often not diagnosed until adolescence).^15^

Plethora of researches was conducted in hopes to find adequate treatment or intervention strategic plans for this rare condition. The treatment of DS is directed according to the specific symptoms that are apparent in each patient and requires the combined efforts of a team of specialists, which include pediatricians, endocrinologists, dermatologists, and other health care professionals.

In fact, treatment of DS is mainly supportive and aims to stabilize the symptoms. For instance, blood glucose levels must be maintained as constantly as possible with the use of frequent or continuous feeds and complex carbohydrates. Furthermore, treatment with recombinant insulin-like growth factor 1 may be considered. A combination treatment with insulin-like growth factor binding protein 3 resulted in an increased lifespan.^16–19^

The prognosis of this devastating disorder is complicated and fatal—whereas the disease onset itself in the prenatal life, usually within the seventh month of gestation; most Donohue Syndrome babies are either aborted or die within the first year of life.^19^

In this study we report a typical case of DS encountered in a 4-month-old child characterized by noticeable phenotypic abnormalities, distinguishing physical features, dysmorphic abnormalities and metabolic symptoms, such as: severe intrauterine and postnatal growth retardation and multiple endocrine dysfunctions. He also fulfilled the biochemical criteria of postprandial hyperglycemia, fasting hypoglycemia, insulin resistance, and hyperinsulinemiain.

Reviewing the literature of *INSR* mutations that are underpinning DS syndrome, over 130 different variations were identified. The majority of these variations were missense and nonsense mutations.^15,1^

The underpinning reasons for the manifestation of the typical phenotypic features especially the endocrine disturbances in DS (hirsutism and hypertrichosis)—encountered also in our patient and usually observed in almost all cases of DS—might be explained by the reduction of sex hormone-binding globulin provoked by insulin action via the type 1 insulin-like growth factor (1 IGF) receptor, which has functional and structural similarities with the insulin receptor.^20^

Type 1 IGF receptors mainly exist during fatal and early postnatal period and their productions from the liver adipocyte cells wither away and disappear during adulthood. As a result of the plummeting activity of sex hormone-binding globulin by insulin action via the type (1 IGF) receptor, augmentation levels of the free circulating androgen (hyperandrogenism) are observed causing the endocrine disturbances in DS regardless of moderately high level of androstenedione, intact level of testosterone and feeble activity of androgens.^21–23^

In addition, many studies demonstrate that IGF-1 acts as autonomous growth hormone and plays important role in body development and have great manipulations on intrauterine linear growth and development.^24–26^

The action of IGF-1 as an essential growth hormone, stimulating linear growth and has self-regulating growth-stimulating effect already at delivery, and throughout babyhood, was proven owing to its deficiency and through the observation of skeleton underdevelopment, as is the case of other organ growth.^27,28^

Indeed, various developmental abnormalities are encountered, including many body organs, such as; heart, brain, weakness and deficit of the body musculature, loss of muscle strength and maturation, facial bones deformities, dwarfism and short extremities as a result of the failure to generate IGF-1.^29–33^

In this unique study, we are stressing the importance of the preventive medicine in rein in the inbreeding mating tradition especially in Mediterranean basin areas, where this phenomenon prevails.

Indeed, our patient was born to first cousin parents from the Arab community in Nazareth, whereas there is a paucity of genetic information on DS. Consanguineous mating is a unique form of union, which may lead to genetic detrimental effects as a consequence of homozygosity of harmful genes. The increased frequency of homozygous genotypes allowed the less common alleles to become manifested homozygous,^34^ thus descendants of consanguineous parents have higher susceptibility to congenital malformation^35,36^ neonatal, postneonatal, and infant mortality than those of unrelated parents.^37–41^

Due to homozygosity of recessive alleles as a result of inbreeding, numerous deleterious alleles appeared in the genome, since the majority of genetic disorders originate from a recessive genotype.^42–46^

The growing propensity for such rare recessive inheritance diseases to appear is triggered by consanguineous marriages, which lead to increased genetic homogeneity of inbred individuals, due to similarities between contributing paternal and maternal genetic material.

**FIGURE 3:**
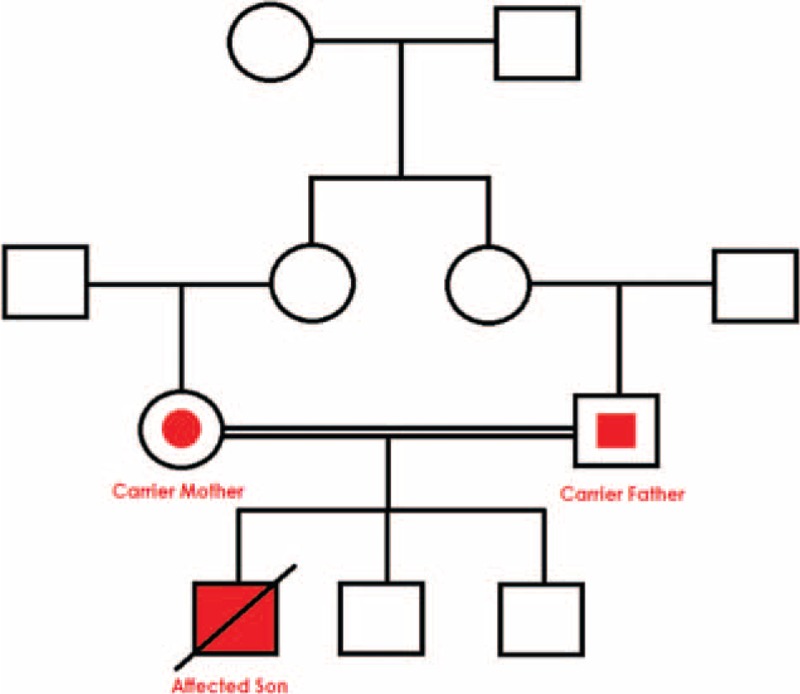
Family pedigree: The family tree shows the affected male infant with leprechaunism, who was homozygous for a mutation in the insulin receptor gene. Both parents, who were first cousins, were heterozygous for the mutant allele and phenotypically and clinically normal.

Historically, since the discovery of the first case by Donohue (1948) to date, the majority of cases encountered in the literature were a result of interbreeding mating (Figure 3), which strengthens the hypothesis that consanguinity is an underlying cornerstone reason for the appearance of these rare genetic disorders.^24,27,47–54^

In our DS case study, the parents are first cousins, and the genetic analysis confirms that they are carriers of a similar mutant allele inherited from a common ancestor. In addition to this, the fact that DS is an uncommon disease and does not occur at high rate in the Arab community suggests that consanguineous mating is a strong trigger for the appearance of such mutations.

Indeed, consanguineous marriage is a particular form of assortative mating, which is deeply seated and deeply rooted in many communities globally; and especially in Mediterranean region.^55^ It is estimated that worldwide over 20% of the human population live in communities with a preference for consanguineous marriage, and over 8.5% of all children have consanguineous parents.

## CONCLUSIONS

There is a scarcity of genetic information on DS among the Arab population. Intermarriages are usually socially motivated but can be genetically harmful. The study and consequences of inbreeding are of considerable concerns in the field of genetics.^4^^2^Due to the harmful consequences of consanguineous mating; this public health phenomenon should be dissuaded and actively discouraged. However, preventive strategy should explain the detrimental consequences of this type of mating. Genetic counseling is extremely recommended and should be a mandatory demand prior to marriage decision. Families at risk should be dissuaded and actively discouraged to have children.
